# Influence of simvastatin on the biological behavior of nucleus pulposus-derived mesenchymal stem cells

**DOI:** 10.22038/IJBMS.2019.14068

**Published:** 2019-12

**Authors:** Zenan Huang, Xiaofei Cheng, Jie Zhao, Zhongjun Liu, Jingcheng Wang, Xinmin Feng, Liang Zhang

**Affiliations:** 1 Department of Orthopaedics, Shanghai Ninth People’s Hospital, Shanghai Jiao Tong University School of Medicine, Shanghai 200011,China; 2 Departmentof Orthopedics, Clinical College of Yangzhou University, Yangzhou 225001, China; 3 Department of Orthopaedics, Peking University Third Hospital, Beijing 100191,China

**Keywords:** Apoptosis, Hypoxia-inducible factor-1α, Intervertebral disc degeneration, Mesenchymal stem cell, Nucleus pulposus, Simvastatin

## Abstract

**Objective(s):**

This research is to study the influences of different concentrations of simvastatin on the biological activities of nucleus pulposus-derived mesenchymal stem cells (NPMSC).

**Materials and Methods:**

NPMSC were cultured with different concentrations of simvastatin (0, 0.01, 0.1, and 1 μM) and assessed to determine the possible effects of simvastatin. The cell proliferation was assessed with CCK-8 assay. The flowcytometry and multilineage differentiation were also performed to identify the stem characterization of the cells. The mRNA expressions of aggrecan, collagen type II, glucose transporter 1 (GLUT-1), vascular endothelial growth factor (VEGF) and hypoxia-inducible factor-1α (HIF-1α) were determined by qRT-PCR.

**Results:**

The results demonstrated that the cells isolated from nucleus pulposus of healthy Sprague-Dawley (SD) rat met the criteria of MSC. NPMSC could form sunflower-like colonies and strongly expressed stem cell-related genes. In addition, NPMSC showed strong ability of chondrogenic, adipogenic and osteogenic differentiation. Simvastatin at certain range concentrations (0.01 μM-0.1 μM)) significantly promoted colony-forming rate and cell proliferation, and inhibited cell apoptosis. Simvastatin could promote expressions of aggrecan, collagen type II, HIF-1α, VEGF and GLUT-1, while 0.1 μmol/l concentration reached the maximum effect. Our study further demonstrated that HIF-1α-intermediated signaling pathway might participate in regulating the biological activities of NPMSC.

**Conclusion:**

Proper concentration of simvastatin can promote the biological behavior of NPMSC, and HIF-1α-intermediated signaling pathway might participate in the mechanism.

## Introduction

Low back pain (LBP) is considered as one of the most common public health problems, causing huge socio-economic cost and human suffering [1-3]. Even though the accurate pathomechanism of LBP stays poorly understood, intervertebral disc degeneration disease (IVDD) is generally accepted as one of the major reasons [4, 5]. However, the current conservative and surgical treatment can only relieve the sufferings rather than reverse the degeneration process or reconstruct the functionality of IVD [6-8].

With the development of the biological strategies in recent years, the biological approaches represented by cell therapy have been widely applied as alternative or complementary treatment of IVD degeneration [9-11]. Accumulating evidence indicated that mesenchymal stem cells (MSC) transplantation is a useful method, which may delay or even reverse IVD degeneration process in some classical IVD degeneration animal models [12-14]. However, the defects of MSC transplantation including cell survival, puncture injury and dysfunction of transplanted stem cells in the harsh microenvironment (high osmolarity, pH, hypoxia, limited nutrition) remain a problem [15-18]. Induction of endogenous stem cell activity and homing of endogenous progenitor cells are being investigated as novel ideas [19, 20]. Damaged tissues themselves will release certain signal molecules to attract or activate progenitor cells to facilitate endogenous cell-mediated tissue repair. The tissue specific ability of endogenous repair has been studied under the background of bone defect, wound healing and myocardial infarction [21-23]. 

Recently, a new type of endogenous MSC was derived from degenerated nucleus pulposus (NP) tissue, and these cells were confirmed to meet the criteria of MSC that was defined by the International Society for Cell Therapy (ISCT) with multilineage differentiation *in vitro* [24]. Studies by San [25] and Liu [26] further identified other types of endogenous progenitor cells from different anatomical regions of IVD. It has been confirmed that NP-derived MSC (NPMSC) could better tolerate high osmolality [27], hypoxia [28] and acidic environment during IVD degeneration [26] compared to other types of MSC. Compared to NP cells, NPMSC has shown better regenerative efficiency though inhibiting the degeneration progress of IVD of rabbit model [29]. But, the declined Tie^2+^ NP progenitor cell population of mouse and human [30], decreased cell activity, colony formation rate and expression of stemness genes in human IVD [31] as well as senescent features [32] suggested that there are still some difficulties in utilizing endogenous progenitor cell for IVD repair.

Simvastatin is commonly applied in control of hyperlipidemia. Tu *et al.* revealed that simvastatin can inhibit extracellular matrix (ECM) degradation and decrease interleukin-1β (IL-1β)-induced NP cell apoptosis [33]. Zhang *et al.* further demonstrated that appropriate concentration of simvastatin could modulate the expressions of collagen type II and proteoglycan partially through upregulating of bone morphogenetic protein-2 (BMP-2) to inhibit mevalonate pathway in NP cells [34]. As to the stem cell, simvastatin can not only promote neural stem cells migration and enhance neuron differentiation rate [35], but also can promote bone marrow-derived MSC osteogenic differentiation and migration *in vitro* [36, 37]. Thus, simvastatin may promote NP cells to secrete ECM and MSC to differentiate. However, the effect of simvastatin on NPMSC is still unknown.

In this study, we speculated that appropriate concentration of simvastatin may promote biological activities of NPMSC. Thus, NPMSC were isolated from normal NP tissue of rat disc, and then flowcytometry and multilineage differentiation were used to detect whether the isolated cells meet the ISCT criteria for MSC. In addition, the influences of various concentrations of simvastatin concerning the biological characteristics of NPMSC were studied *in vitro*, including cell apoptosis, cell proliferation, cell colony-forming and functional genes expressions. 

## Materials and Methods

*Cell isolation and culture*

All procedures performed for the present work were authorized by the Institutional Animal Use and Care Committee of the Clinical Medical College of Yangzhou University, Yangzhou, China. Six Sprague-Dawley (SD) rats (age, 4-6 months; weight, 300-400 g) were obtained from Jiangsu University Animal Center (License No. SCXK (Su) 2013-0011). After euthanasia by injecting 10% chloral hydrate intraperitoneally, the NP tissues were obtained under aseptic conditions. The isolated NP tissues were digested by collagen type II (Gibco, USA) and then trypsin (Gibco, USA), and then washed twice with phosphate-buffered saline (PBS) and centrifuged. After that, the pellets were suspended with F12 medium with 1% penicillin (Gibco, USA) and 20% fetal bovine serum (FBS) (Hyclone, USA). Lastly, plated cells (1×10^5^ cells/mL) were incubated in cell incubator. The medium replaced every 3 days and observed with microscope. The adherent cells were harvested and subcultured at 1: 3 when 80% confluence reached.

*Characteristics of NPMSC proliferation curve*

The proliferation capacity of the third generation of NPMSC was measured with CCK-8 (Dojindo, Tokyo, Japan). Briefly, P3 NPMSC at 1×10^3 ^cells/well were cultured in 96-well plates. At the corresponding time after initial plating, 10 μl reagents were added in each well and placed in incubator for 3 hours. Optical density (OD) values were measured with microplate reader at 450 nm (Bio-Rad, Hercules, USA). 

*Immunophenotype of NPMSC by flowcytometry*

For the immunophenotypic characterization, P3 NPMSC were trypsinized, collected, and resuspended with PBS. Then, according to the ISCT recommendation, the cells were incubated with monoclonal antibodies: CD105-FTTC, CD90-FTTC, CD73-FTTC, CD45-PE, and CD11b (Bioscience, USA) in the dark for 30 minutes. Isotype IgG control antibodies (BDB) (Bioscience, USA) were served as controls. After that, the cells were resuspended with PBS, which contained 1% paraformaldehyde (Sangon Biotech, China). The fluorescence intensity of each monoclonal antibody and the percentage of positive cells were examined using flowcytometry (BD, USA).

*2.4. Multilineage differentiation of NPMSC*

*. Osteogenic differentiation*

NPMSC were cultured in 6-well plate with osteogenic differentiation medium (medium containing 87.5% basal media, 10% FBS, 1.0% penicillin-streptomycin, 1.0% glutamine, 1.0% β-glycerophosphate, 0.2% ascorbate and 0.01% dexamethasone) according to 2.0×10^4^ cells/ml [38]. The medium was changed every 3 days. Through 21 days differentiation, the cells were fixed and stained with alizarin red and then observed under inverted microscope.

*Chondrogenic differentiation*

NPMSC were cultured in 6-well plate according to 3.0×10^5^ cells/ml and incubated with chondrogenic differentiation medium (medium containing 97.0 basal media, 1.0% transforming growth factor beta-3 (TGF-β_3_), 0.01% dexamethasone, 0.1% sodium pyruvate, 0.3% ascorbate, 0.1% proline and 1.0% insulin-transferrin-selenium (ITS) supplement) for 28 days [38]. The cells were embedded with paraffin and cut into slices. Then, the slices were stained with alcian blue and observed though inverted microscope. 

*Adipogenic differentiation*

NPMSC were cultured in 6-well plate according to 2.0×10^4^ cells/ml at 37°C with 5% CO_2_. Adipogenic differentiation medium A (medium containing 87.5% basal media, 0.1% IB-MX (3-isobutyl-1-methylxanthine), 1.0% glutamine, 1.0% penicillin-streptomycin, 0.1% dexamethasone, 0.1% rosiglitazone, 0.2% insulin and 10% FBS) was added and incubated for 3 days. Then, medium B was (medium containing 87.5% basal media, 0.2% insulin, 1.0% penicillin-streptomycin, 1.0% glutamine and 10% FBS) changed and incubated for 1 day [38]. This 4-day cycle was repeated four times and then incubated with medium B for 7 days. After that, the cells were fixed and stained by oil red O and then observed with inverted microscope.

*Expressions of stem cell-related genes*

The mRNA expressions of Sox-2, Oct-4 and Nanog were assessed by qRT-PCR and compared to those of BMSC (gifted from Pro. Zhao’s lab). Briefly, total RNA was extracted with RNA extraction kit (Carlsbad, CA, USA). The values were compared to glyceraldehyde-3-phosphate dehydrogenase (GAPDH) and the relative genes expressions were calculated through the comparative Ct method. The information of primer sequences (synthesized by Invitrogen) were shown in Table 1. 

*The influence of simvastatin on th*e *characteristic of NPMSC*

*Preparation of different concentrations of simvastatin culture medi* *um*

Different concentrations of simvastatin culture medium (Sigma st. Louis, MO, USA) were prepared with Hatano’s method [39]. Culture medium of four concentration levels 0 μM, 0.01 μM, 0.1 μM and 1 μM were obtained. 

*CCK-8 assay* NPMSC (1,000 cells/well) were plated in 96-well plates with different concentrations of simvastatin media. Then, samples were harvested at 1, 3, 7, 11 and 15 days; 10 μl reaction solution was added and then cultured 3 hrs. OD values were measured through microplate reader at 450 nm.

**Table 1:** Primer sequences used for this study.

**Gene name**	**Forward primer (5'-3')**	**Reverse primer (5'-3')**
*Sox-2* *(EU661361.1)*	GGCAATCAAATGTCCATT	GTCTGTAACGGTCCTTAA
*Nanog* *(JQ801747.1)*	GATATGGTGGCTCCTCTC	AGGCTGGTCTTGAACTTAT
*Oct-4* *(HQ907734.1)*	AGCAGATCACTAGCATTG	TCATACTCTTCTCGTTGG
*Aggrecan* *(J03485.1)*	CCCAAACAGCAGAAACAGCC	AGCTGGTAATTGCAGGGGAC
*Type II collagen* *(X16711.1)*	ATTCCTGGTGAAGCTGGAGC	AGCGTCACCTTTCTGTCCAG
*HIF-1α* *(JQ308830.1)*	GTCTCCATTACCTGCCTCTG	GATTCTTCGCTTCTGTGTCTTC
*VEGF* *(KJ729036.1)*	TCTTGTCTCTGGCGTGTTCC	TGCTGAGGTAACCTGTGCTG
*GLUT-1* *(S77924.1)*	GTTCCTTCTCTGTGGGCCTC	CGGAACAGCTCCAAGATGGT
*GAPDH* *(KC205035.1) *	TGACTTCAACAGCGACACCCA	CACCCTGTTGCTGTAGCCAAA

**Figure 1:**
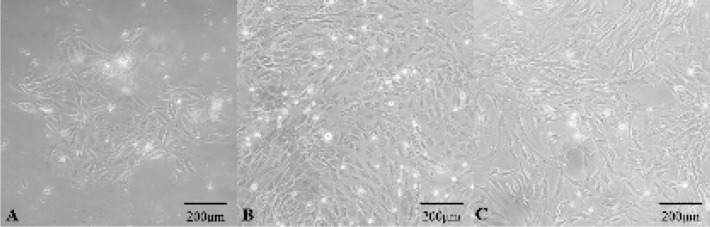
Morphologies of NPMSC cultured *in vitro*. (A): Adherent cells presented sunflower-like clonal growth in the first generation. (B): The passaged cells grew in mainly spindle-shaped. (C): The morphology of P3 cells was basically spindle shape. NPMSC: Nucleus pulposus-derived mesenchymal stem cells.

**Figure 2:**
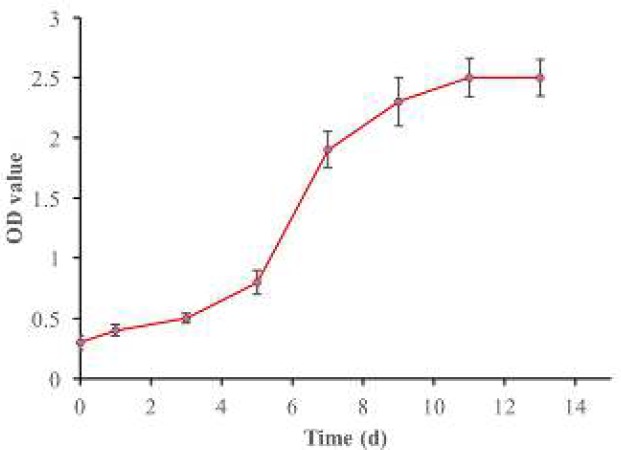
The growth curve of NPMSC. The growth curve showed that NPMSC proliferated slowly in the initial 3-5 days, presented the logarithmic growth phase in 8-13 days, and then reached cell growth plateau in 11-13 days. NPMSC: Nucleus pulposus-derived mesenchymal stem cells.

**Figure 3:**
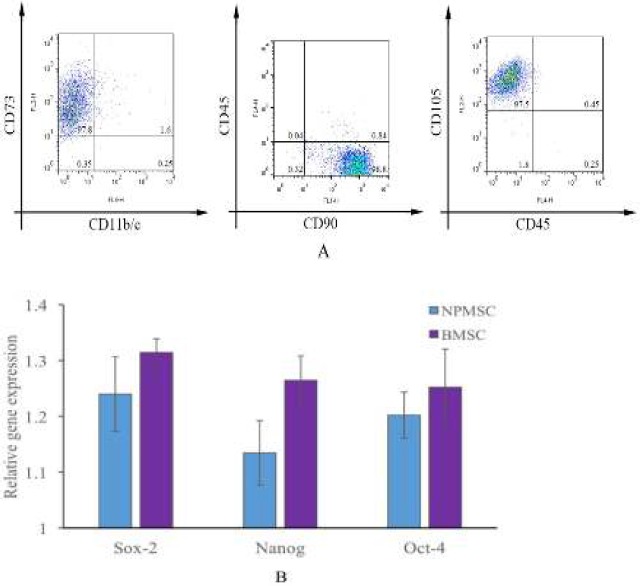
Immunophenotypic analysis and expressions of stem cell related genes. (A): Immunophenotypic analysis of cell surface markers. NPMSC were highly positive for CD105, CD73 and CD90 and negative for CD11b/c and CD45. (B): The expressions of Sox-2, Oct-4 and Nanog of NPMSC were comparable to those of BMSC (*P*> 0.05). NPMSC: Nucleus pulposus-derived mesenchymal stem cells.

**Figure 4:**
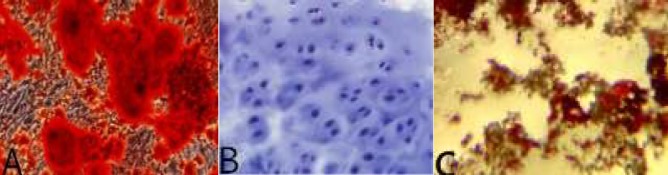
Differentiation staining results of NPMSC: (A): Alizarin red staining; (B): Alcian blue staining; (C): Oil red O staining. NPMSC: Nucleus pulposus-derived mesenchymal stem cells.

**Figure 5:**
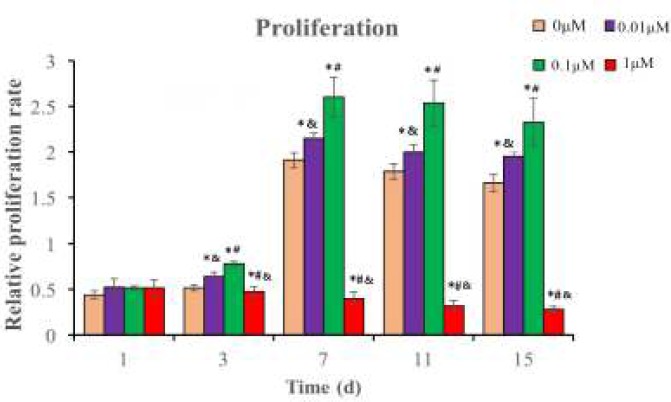
The relative proliferation rate of NPMSC incubated with different concentrations of simvastatin. The Figures are expressed as means ± SD, n = 3. *P<0.05 indicated significant difference compared to 0 μM group, ^#^*P*<0.05 indicated significant difference compared to 0.01 μM group, ^&^*P*<0.05 indicated significant difference compared to 0.1 μM group. NPMSC: Nucleus pulposus-derived mesenchymal stem cells.

**Figure 6:**
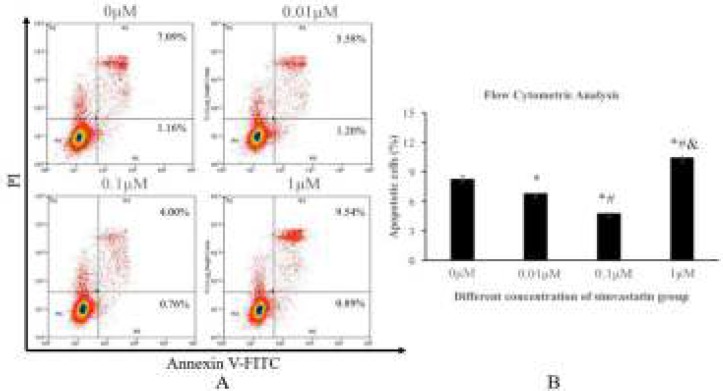
The apoptotic cells rates of NPMSC incubated with different concentrations of simvastatin for 3 days. (A): Annexin V-positive/PI-negative population of NPMSC with different concentrations of simvastatin. (B): Flowcytometric analysis of apoptotic rate. The data are expressed as means ± SD, n = 3. *P<0.05 indicated significant compared to 0 μM group, ^#^*P*<0.05 indicated significant difference compared to 0.01 μM group, ^&^*P*<0.05 indicated significant difference compared to 0.1 μM group. NPMSC: Nucleus pulposus-derived mesenchymal stem cells.

**Figure 7:**
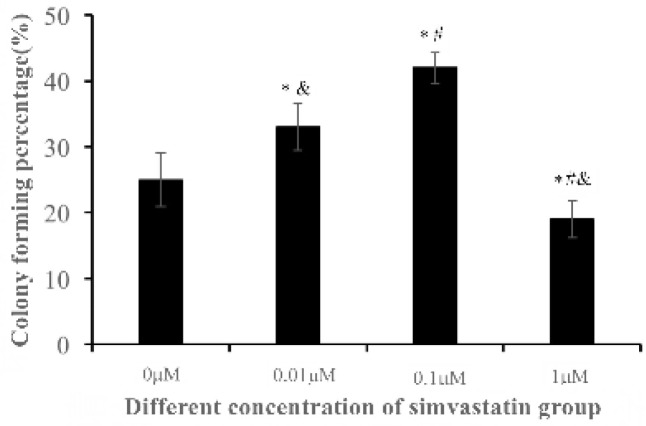
The colony-forming rate of NPMSC cultured with different concentrations of simvastatin for 3 days. The data are expressed as means ± SD, n = 3. *P<0.05 indicated significant difference compared to 0 μM group, ^#^*P*<0.05 indicated significant difference compared to 0.01 μM group, ^&^*P*<0.05 indicated significant difference compared to 0.1 μM group. NPMSC: Nucleus pulposus-derived mesenchymal stem cells.

**Figure 8:**
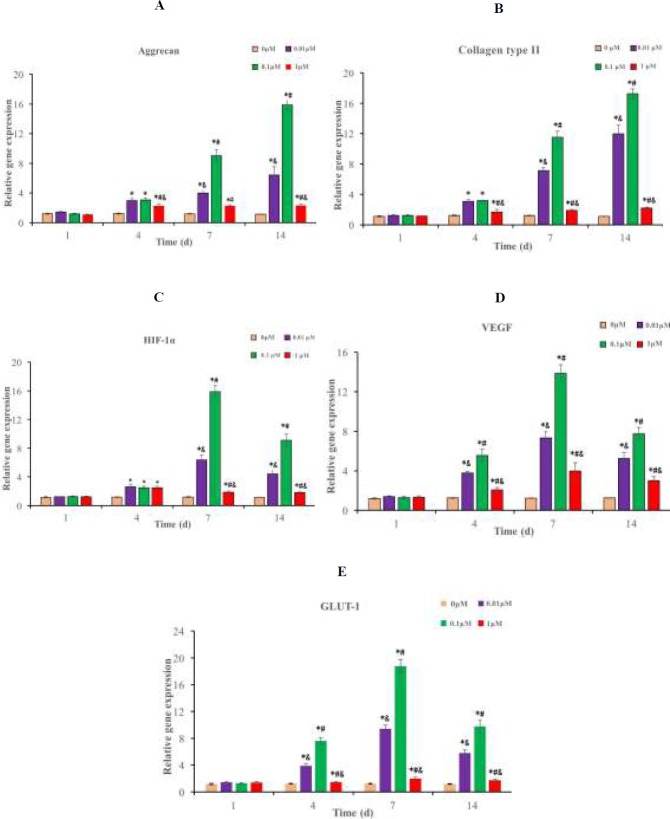
mRNA expressions of related genes of NPMSC cultured with different concentrations of simvastatin for 3 days. The Figures are described as means ± SD, n = 3. (A): aggrecan expression; (B): collagen type II expression; (C): HIF-1α expression; (D): VEGF expression; (E): GLUT-1 expression. *P<0.05 indicated significant difference compared to 0 μM group, ^#^*P*<0.05 indicated significant difference compared to 0.01 μM group, ^&^*P*<0.05 indicated significant difference compared to 0.1 μM group. NPMSC: Nucleus pulposus-derived mesenchymal stem cells, GLUT-1: Glucose transporter 1, VEGF: Vascular endothelial growth factor, HIF-1α: Hypoxia-inducible factor-1α .

*Apoptosis assay of NPMSC*

NPMSC (1×10^5^ cells/ml) were seeded onto 6-well plates and incubated with different concentrations of simvastatin for 3 days. Cell death and apoptosis were detected with flowcytometry using propidium iodide (PI) and Annexin V-FITC (Invitrogen, USA) for observing the influence of simvastatin on apoptosis. The cells were resuspended and cultured with PI (Keygen, Biotech, China) and Annexin V-FITC for 15 mins. The Annexin V-positive/PI-negative cells were apoptotic. The Cell Quest software (Biosciences, USA) was used to assay.

*Colony-Forming Assay*

NPMSC (1,000/cm^2^) were seeded onto 6-well plates and incubated with different concentrations of simvastatin media. After incubating 2 weeks, cells were fixed with 4% paraformaldehyde for 15 min. The cells were then stained 15 min with crystal violet (0.1%) (Keygen Biotech, Nanjing, China). Cell colonies that contained more than 100 cells were recorded and the colony-forming rates were counted as the colonies numbers divided by the initial adherent cell numbers.

*The mRNA expressions of related genes*

NPMSC (1×10^5^ cells/mL) were plated in 6-well plates and cultured with different concentrations of simvastatin media at 37°C with 5% CO_2_ for 1, 4, 7 and 14 days. The influence of simvastatin on the expressions of hypoxia-inducible factor-1α (HIF-1α), aggrecan, collagen type II, glucose transporter 1 (GLUT-1) and vascular endothelial growth factor (VEGF) was detected by qRT-PCR. Quantities of gene expression were calculated and compared to GAPDH. 

*Statistical analysis* The experimental figures were described as mean ± SD and analyzed with SPSS (18.0) software (IBM, USA). Univariate general linear analysis and one-way analysis of variance (ANOVA) were used to assay the differences among different groups. *P* values < 0.05 indicate statistically significant. 

## Results

*Cell morphology*

After 3-5 days of initial culture, adherent cells presented sunflower-like colonal growth in the first generation as shown in Figure.1A, and then the adherent cells gradually increased after 5-7 days. The adherent cells needed approximately 3-4 weeks to reach 80%-90% confluence. When cells were passaged, the cell growth was significantly faster than primary cells, and the cells grew in mainly spindle-shaped (Figure.1B). P3 cells morphology was basically spindle shape (Figure.1C). 

*Analysis* *of c* *ell viability and proliferation*

The growth curves determined by CCK-8 assay showed that NPMSC (1,000 cells/well) proliferated slowly in the initial 3-5 days and then presented logarithmic growth phase in 8-13 days and then reached cell growth plateau in 11-13 days (Figure.2).

*Immunophenotype analysis and expressions of stem cell-related genes*

NPMSC highly expressed CD73 (97.8%), CD90 (98.8%) and CD105 (97.5%) and were negative for CD11b/c and CD45 (Figure.3A). The expression levels of commonly expressed stem cell genes (Oct-4, Nanog and Sox-2) in NPMSC were comparable to those in BMSC (Figure.3B, *P* > 0.05). 

*Different lineage differentiation of NPMSC*

Alizarin red staining result for 21 days’ osteogenic induction indicated that NPMSC was stained red and calcium deposits could be identified (Figure.4A). NPMSC was also stained with alcian blue after chondrogenic induction for 28 days (Figure.4B). After adipogenic induction, oil red O staining result demonstrated that NPMSC was stained red and lipid formation was found (Figure.4C). 

*The* *influence* *of simvastatin on the* *biological* *characteristics* *of NPMSC*

*Proper concentrations of simvastatin promote cell proliferation*

The proliferation of NPMSC significantly increased in the 0.01 μM (2.14 ± 0.41) and 0.1 μM groups (2.67 ± 0.24) (*P* < 0.05), which reached the peak on the 7th day. The proliferation rate of 0.1 μM group (2.67 ± 0.24) was higher than that of 0.01 μM group (2.14 ± 0.41) (*P* < 0.05). However, the proliferation rate decreased with time at the 1.0 μM group (0.47 ± 0.32) (*P* < 0.05) as shown in Figure.5.

*Proper concentrations of simvastatin decrease cell apoptosis*

The percentage of apoptotic cells decreased in the 0.01 μM (6.92 ± 0.85) and 0.1 μM groups (5.13 ± 0.91) compared to that of 0 μM group (8.64 ± 0.89) significantly (*P* < 0.05), and the apoptotic cell rate of 0.1 μM group was higher than that of 0.01 μM group (*P <0.05*). However, the percentage of apoptotic cells significantly increased in the 1 μM group (10.89 ± 1.13) compared to that of 0 μM group (8.64 ± 0.89) (*P* < 0.05) as shown in Figure.6. 

*Proper concentrations of simvastatin promote cell* *colony-forming ability *

NPMSC cultured with different concentrations of simvastatin showed significant colony-forming abilities. The colony-forming rate of both 0.01 μM (33.76 ± 0.54) and 0.1 μM groups (42.34 ± 0.88) significantly increased compared to that of 0 μM group (24.36 ± 0.63), and the colony-forming rate of 0.1 μM group was even higher than that of 0.01 μM group (*P* < 0.05). However, the colony-forming rate of 1 μM group (19.84 ± 0.47) decreased compared to that of 0 μM group (*P* < 0.05) (Figure.7). 

*Proper concentrations of simvastatin increase related mRNA expressions*

The mRNA expressions of collagen type II and aggrecan increased gradually at different concentrations of simvastatin group from the 4th day and reached the peak on the 14th day. The highest mRNA expressions of aggrecan (17.24 ± 0.62) and collagen type II (17.83 ± 0.56) of 0.1 μM group were greater than those of 0.01 μM (5.96 ± 0.82 and 12.83 ± 0.53) and 1 μM groups (2.83 ± 0.59 and 2.13 ± 0.41) (*P* < 0.05) (Figure.8A-B). The mRNA expressions of VEGF, HIF-1α and GLUT-1 in NPMSC began to increase from the 4th day and reached the peak at the 7th day (Figure.8C-E). The highest mRNA expressions of 0.1 μM group (HIF-1α 17.28 ± 0.92, VEGF 13.93 ± 0.74 and GLUT-1 19.28 ± 0.96) were higher than those of 0.01 μM (6.93 ± 0.43, 7.64 ± 0.47 and 9.32 ± 0.69) and 1 μM groups (2.13 ± 0.21, 4.13 ± 0.64 and 2.15 ± 0.28) (*P* < 0.05).

## Discussion

IVD was believed as a non-renewable organ in previous studies. However, the results of recent researches demonstrated that MSC can be isolated from NP tissues [25, 26]. NPMSC shares the same morphological characteristics, cell phenotype and stem cell-related gene expressions as MSC. All of our findings indicated that the cells isolated from NP tissues fulfilled the ISCT requirement of MSC definition. NPMSC could not only be isolated from healthy and degenerate NP tissue but can also be capable to adjust to the harsh microenvironment of IVD [31, 32, 40]. Thus, inducing endogenous NPMSC to repair and reconstruct the function of degenerative IVD may be the future direction of IVDD regeneration.

Statins are commonly-prescribed drugs used to inhibit hyper-cholesterolemia in clinical use. In addition, they can strengthen the function and other biological activities of MSC to enhance the therapeutic effect [36, 37, 41]. The influences of simvastatin on IVD cells have been verified, in which simvastatin were mostly confirmed to promote the expressions of collagen type II, BMP-2 and aggrecan [33, 34]. Zhang *et al.* demonstrated that simvastatin (0.3 μM) can not affect NP cells viability; however, cell viability significantly decreased when the dose increased to 1 μM and 3 μM [34]. In addition, Tu *et al.* demonstrated that simvastatin (5-50 μM) did not induce significant cytotoxicity to the co-cultured NP cells, but can inhibit IL-1β induced cell apoptosis [33]. However, there is only few information about statins’ effect on MSC, as higher dose of fluvastatin (10 μM) can induce morphological changes [42], lower dose (1 μM) and medium dose (5 μM) of simvastatin significantly reduce cell proliferation [41]. Whether simvastatin has similar effect on NPMSC is still unknown. 

Thus, in our present work, NPMSC was cultured with different concentrations of simvastatin to observe its effects on the biological properties of NPMSC. Our data suggested that proper concentrations of simvastatin could promote the proliferation and colony-forming ability of NPMSC in a time-dose dependent manner. The function of the healthy IVD depends on the ECM integrity of the IVD, which mainly consist of collagen and proteoglycans. The use of NPMSC in the biological treatment for IVDD is to differentiate into NP cells. In the present study, the main ECM of normal NP cells were regarded as collagen type II and aggrecan. The results of qRT-PCR showed that the expressions of collagen type II and aggrecan increased in the 0.01 μM and 0.1 μM groups to a certain extent in a time-dose dependent manner. All the above results demonstrated that simvastatin with the concentrations of 0.01 μM and 0.1 μM could not only decrease NPMSC apoptosis but also promote ECM synthesis and cell proliferation. Thus, proper concentrations of simvastatin can induce NPMSC differentiate into NP cells and secrete ECM, which may be helpful in the maintenance of IVD stability and may reverse the degenerative process. However, 1.0 μM group would decrease cell proliferation and promote cell apoptosis rate, which may be attributed to the cytotoxic effects of high concentration of simvastatin.

Some studies have found that multiple types of MSC from bone marrow, placenta, fat, and ligamentum flavum can be differentiated to NP cells under hypoxia [43-46]. HIF-1α is an important transcriptional regulator under hypoxia, which affects cell proliferation, apoptosis and differentiation. Cui *et al.* found that local application of simvastatin can promote migration and homing of BMSC through upregulating the expressions of HIF-1α and BMP-2 to promote bone defect repair [47]. It is commonly known that persistent glucose supply is important for cell proliferation and secretion of ECM. Park *et al.* also found that HIF-1α participates in the metabolism of adipose-derived MSC and glucose uptake through increased expressions of GLUT-1 and GLUT-3 [48]. Li *et al.* further demonstrated that HIF-1α promote NPMSC proliferation and chondrocyte differentiation by upregulating the expressions of its downstream genes GLUT-3, GLUT-1 and VEGF [28]. VEGF can improve MSC viability and the influence of MSC transplantation in the ischemic diseases treatment [49, 50]. Chen *et al.* found that certain range of simvastatin (0.001-0.100 μM) could enhance the proliferation and secretion ability of BMSC by upregulating the expression of VEGF [51]. Tamama *et al.* further demonstrated that VEGF can enhance the secretions of growth factor and proliferation of BMSC [52]. Our study is the first to demonstrate that different concentrations of simvastatin could increase the expressions of VEGF, GULT-1 and HIF-1α in NPMSC, and the expressions increased along with increasing concentrations of simvastatin from 0.01 μM to 0.1 μM. These results suggested that HIF-1α-mediated signaling could participate in the simvastatin-dependent response of NPMSC.

## Conclusion

In summary, our results indicated that proper concentration of simvastatin is a favorable factor to promote the biological behavior of NPMSC, and this promoting effect may occur via HIF-1α-mediated signaling pathway. Our study will lay a foundation to further investigate the mechanism of simvastatin-based therapy for interverbal disc degeneration. 
